# Acknowledgement

**Published:** 1862-12

**Authors:** 


### ACKNOWLEDGEMENT.

We are happy to acknowledge the receipt, from Dr. Wood, of a lot of his Plastic Metallic Filling, for the use of the class in the Dental College this winter, for which he has the best thanks of all concerned. Let others do likewise. T.

